# Whole body metabolic tumor volume is a prognostic marker in patients with newly diagnosed stage 3B non-small cell lung cancer, confirmed with external validation

**DOI:** 10.1186/s41824-017-0013-z

**Published:** 2017-12-01

**Authors:** Brittany Z. Dashevsky, Chenpeng Zhang, Li Yan, Cindy Yuan, Lingyun Xiong, Yongmei Liu, Haiyan Liu, Feng-Ming Spring Kong, Yonglin Pu

**Affiliations:** 1 Department of Radiology, University Of Chicago, 5841 S. Maryland Ave. MC 2026, Chicago, IL 60637 USA; 2 Department of Nuclear Medicine, RenJi Hospital, School of Medicine, Shanghai Jiao Tong University, Shanghai, China; 3 Department of Radiation Oncology, Linyi People’s Hospital, Linyi, Shandong China; 4 Department of Cell Biology, Shandong University, Jinan, Shandong China; 5 Department of Thoracic Oncology, China West University Hospital, Shichuan, Chengdu, China; 6 Department of Nuclear Medicine, First Hospital of Shanxi Medical University and Molecular Imaging Precision Medical Collaborative Innovation Center, Shanxi Medical University, Taiyuan, 030001 Shanxi China; 7 Radiation Oncology, Medical and Molecular Genetics, Indiana University School of Medicine, Indianapolis, USA; 8 Department of Radiation Oncology, Simon Cancer Center, Indiana University School of Medicine, 535 Barnhill Drive, Indianapolis, IN 46202 USA

**Keywords:** Lung cancer, Tumor volume, FDG, PET/CT

## Abstract

**Purpose:**

TNM Stage 3B encompasses a wide range of primary tumor and nodal metastatic tumor burden. This study aimed to evaluate the prognostic value of quantitative FDG PET/CT parameters in patients with newly diagnosed Stage 3B Non-Small Cell Lung Cancer (NSCLC).

**Materials and Methods:**

Institutional review board approved retrospective study identified patients diagnosed with Stage 3B NSCLC (8^th^ edition TNM classification) on baseline FDG PET/CT at two medical centers (Medical centers A and B), between Feb 2004 and Dec 2014. Patients were excluded if they had prior NSCLC treatment or recent diagnosis of a second primary cancer. Quantitative FDG PET/CT parameters including whole body metabolic tumor volume (MTVwb), total lesion glycolysis (TLGwb), and maximum standardized uptake value (SUVmaxwb) were measured from baseline PET/CT using Edge method with Mimvista software. The primary endpoint was overall survival (OS). Cox proportional hazard regression and Kaplan-Meier overall survival analyses were used to test for an association between OS and quantitative FDG PET/CT parameters. The distributions of MTVwb, TLGwb, SUVmaxwb were skewed, so a natural logarithm transformation was applied and the transformed variables [(ln(MTVwb), ln(TLGwb), and ln(SUVmaxwb)] were used in the analysis.

**Results:**

The training set included 110 patients from center A with Stage 3B NSCLC. 78.2% of patients expired during follow-up. Median OS was 14 months. 1-year, 2-year, and 5-year OS was 56.5%, 34.6% and 13.9%, respectively. Univariate Cox regression analysis showed no significant difference in OS on the basis of age, gender, histology, ln(TLGwb), or ln(SUVmaxwb). ln(MTVwb) was positively associated with OS [hazard ratio (HR) of 1.23, *p* = 0.037]. This association persisted on multivariate Cox regression analysis (HR 1.28, *p* = 0.043), with adjustments for age, gender, treatment and tumor histology. External validation with 44 patients from center B confirmed increasing MTVwb was associated significantly worse OS. An MTVwb cut-off point of 85.6 mL significantly stratified Stage 3B NSCLC patient prognosis.

**Conclusion:**

MTVwb is a prognostic marker for OS in patients with Stage 3B NSCLC, independent of age, gender, treatment, and tumor histology.

## Introduction

Lung cancer is the leading cause of cancer death within the United States (Siegel et al., 2016). The TNM staging system developed by the American Joint Committee on Cancer (AJCC)/Union for International Cancer Control (UICC) is the primary means for classifying patient disease status and predicting patient prognosis, with the most recent 8^th^ edition recently introduced by the International Association for the Study of Lung Cancer (IASLC) (Detterbeck et al., 2016; Asamura et al., 2015; Rami-Porta et al., 2015; Goldstraw et al., 2016). Stage 3B Non-small Cell Lung Cancer (NSCLC) now comprises patients without distant metastases (M0) who have stage T3 or T4 primary disease, with ipsilateral mediastinal and/or subcarinal nodal metastases (N2), as well as patients with T1 or T2 primary disease with metastases in scalene, supraclavicular, contralateral mediastinal or contralateral hilar lymph nodes (N3). In other words, Stage 3B encompasses a wide range of primary tumor burden, including patients with primary tumors ranging from <1 cm (T1a) to ≥7 cm (T4) in greatest dimension and N0 to N3 nodal metastases.

For patients with locally advanced lung cancer, fluorodeoxyglucose (FDG) positron emission tomography/computed tomography (PET/CT) enables detection of CT-occult, metabolically active tumor. Maximum standardized uptake value (SUVmax) is an established measurement of maximal tumor metabolic activity and facilitates identification of tumor metastases, but it fails to take into account total tumor abundance/burden. Quantitative FDG measurements, namely the amount of whole body metabolic tumor volume (MTVwb) and total lesion glycolysis (TLGwb), may be used to quantify both total tumor burden and metabolic activity. These parameters have been found to be associated with OS for both surgical and non-surgical NSCLC patients, after adjusting for clinical stage (Liao et al., 2012a; Zhang et al., 2013a; Zhang et al., 2013b). These PET/CT metrics have yet to be evaluated for Stage 3B NSCLC patients (Ohri et al., 2015).

Patients with Stage 3B NSCLC are rarely surgical candidates due to mediastinal invasion or extensive nodal disease, and are routinely treated with combined radiation and chemotherapy, though options are constantly evolving to cater to a more individualized patient-centered therapeutic approach (Rocco et al., 2016). The overall survival (OS) of Stage 3B NSCLC patients at 2 and 5 years is 44% and 26%, respectively (Goldstraw et al., 2016). If we can identify additional prognostic markers for these patients, we may be able to better stratify Stage 3B NSCLC patients, and determine who is more likely to benefit from more aggressive therapy.

Here we aimed to evaluate the prognostic value of MTVwb, TLGwb, and SUVmaxwb, on baseline FDG PET/CT in patients with Stage 3B NSCLC.

## Materials and methods

This Health Insurance Portability and Accountability Act compliant retrospective study received institutional review board approval. The need for informed consent was waived.

### Patient cohort

Utilizing a retrospective search of our health information system, patients were included if they met both of the following inclusion criteria: a. diagnosed with Stage 3B NSCLC in accordance with the 8^th^ edition of the TNM classification system; and b. received baseline staging PET/CT between February 2004 and Dec 2014. Patients were excluded if they either: 1. received prior lung cancer treatment, including chemotherapy, radiation, or surgery; or 2. had a second primary cancer diagnosed from 5 years before to 2 months after the FDG PET/CT scan conducted for NSCLC diagnosis and staging. 110 consecutive patients were identified from center A and served as the training set. The external validation set included 44 patients managed and imaged at institution B from 2008 to 2014. These patients received a FDG PET/CT within 3 months of starting radiation therapy.

Patient demographics, clinical information, and follow-up were collected from the health information system. Patients were censored at date of last contact.

### PET/CT acquisition

Imaging was performed on a Siemens mCT scanner (Siemens Healthcare, Knoxville, Tenn) or Reveal HD scanner (CTI, Knoxville, Tenn). All patients fasted for a minimum of 4 h prior to imaging, confirmed by fingerstick serum blood glucose levels <200 mg/dl. Patients were injected with 370-555 MBq ^18^F–FDG. At center A (the training dataset), whole body PET/CT imaging was acquired approximately 90 min later. At center B (the validation dataset), patients were scanned approximately 60 min later. Concomitant low-dose non-contrast or diagnostic CT extending from the skull base to thighs was performed for anatomic localization and attenuation correction.

**Fig. 1:**
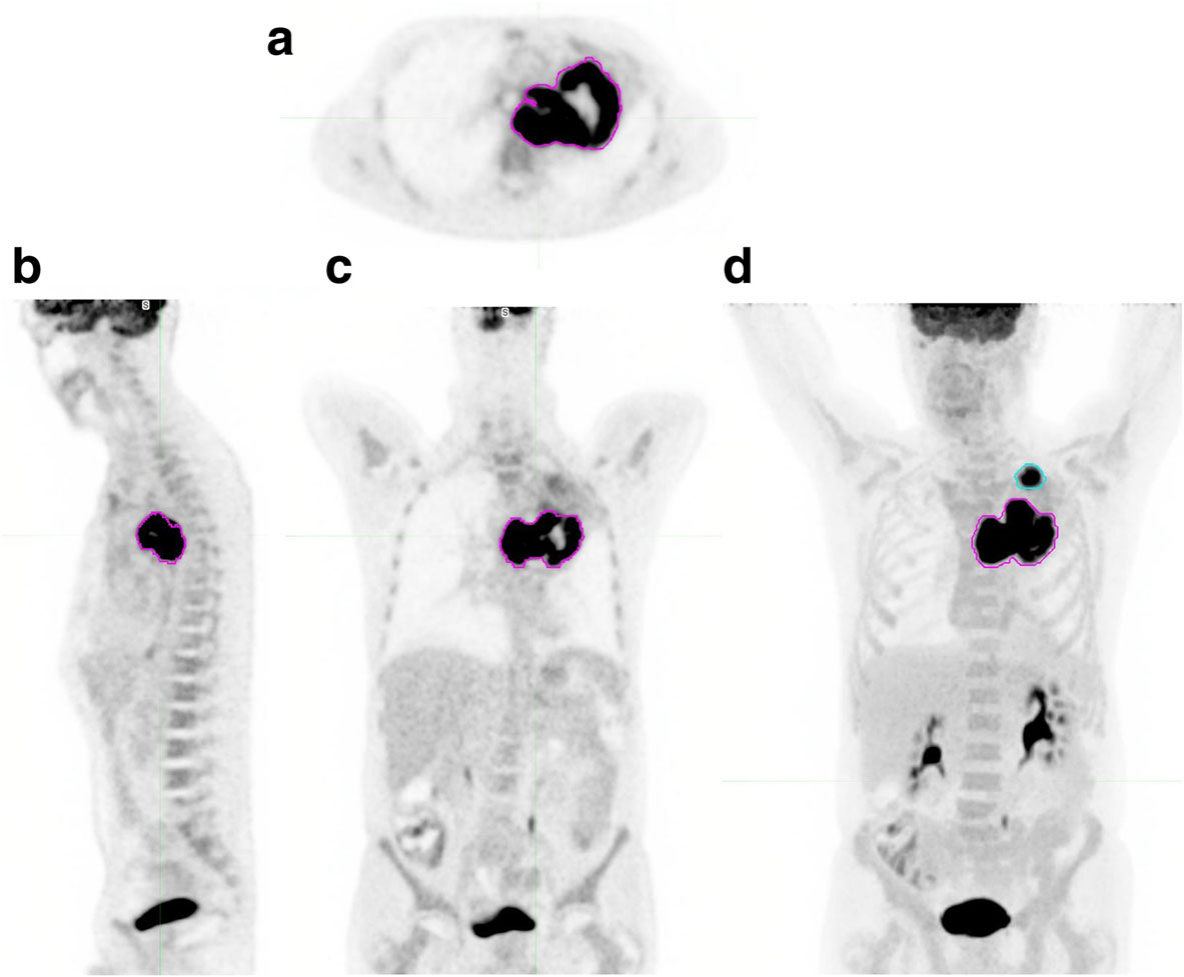
Whole-body PET images of a 59 year-old male with newly diagnosed squamous cell carcinoma, stage IIIB (T4N2M0). This study was performed for initial staging. Colored regions of interest (ROIs) show the tumor contours. The MTVwb in mL is the sum of tumor volumes in all ROIs. Axial image (**a**), sagittal image (**b**), coronal image (**c**) and maximal intensity projection image (**d**)

PET/CT tumor volume, SUVmaxwb, MTVwb, and TLGwb were measured using the PET Edge tool of MIMvista software as illustrated in Fig. 1, using semi-automated tumor segmentation, as previously described (Liao et al., 2012a).

Briefly, SUVmax for each tumor lesion was defined as: $\frac{\mathrm{decayed}\  \mathrm{corrected}\  \mathrm{activity}/\mathrm{tissue}\  \mathrm{volume}}{\mathrm{injected}\  \mathrm{dose}/\mathrm{injected}\  \mathrm{weight}}$. SUVmaxwb was taken to be the largest SUVmax among all tumor lesions identified on baseline staging PET/CT. MTVwb was defined as the total segmented volume of all FDG-avid tumors in the body. TLG for a single tumor lesion is the product of MTV and the average SUV for the lesion. TLGwb was calculated as the sum of the TLGs of all segmented tumor.

### Statistical analysis

Statistical analyses were performed using Stata Software version 14. OS was considered the primary endpoint and defined as the time from baseline PET/CT to time of any cause mortality. Analysis was conducted using natural-logarithm transformed values of the PET parameters [(ln(MTVwb), ln(TLGwb), and ln(SUVmaxwb)] in order to achieve a near-normal distribution of independent variables. Univariate and multivariate analyses were performed with Cox proportional hazard regression models to test for an association with the primary end point, OS. *P*-value of <0.05 from a two-tailed test was considered significant. Survival curves were plotted using the Kaplan-Meier method.

## Results

### Model fitting

The model was fitted using the training dataset of 110 patients with Stage 3B NSCLC (41% adenocarcinoma; 32% squamous cell; 27% other histologic sub-type) who met the inclusion criteria, with patient characteristics summarized in Table 1. The median patient age was 67.3 (range 31.4–83.9) years. There were 47 Caucasian patients, 60 African American patients and 3 Asian patients. The majority of patients (69%) were managed non-surgically (with chemotherapy and/or radiation), while 22% received surgical treatment, and 9% did not treatment. 86/110 patients expired during follow-up. The median OS was 14 months. 1-year, 2-year, and 5-year OS was 56.5%, 34.6% and 13.9%, respectively. The median follow-up among survivors was 26.0 months (inter-quartile range 13.6 to 57.1 months).

**Table 1:** Characteristics of patients from the University of Chicago

Clinical and PET Parameters	Number of patients	Percentage
Gender		
Male	47	43%
Female	63	57%
Race		
Caucasian	47	43%
African American	60	55%
Asian	3	3%
Treatment		
Surgically	24	22%
Non-surgically	76	69%
None	10	9%
Histological subtype		
Adenocarcinoma	45	41%
Squamous cell	35	32%
Other types^a^	30	27%
Age (Median and range, years)	67.3(31.4–83.9)	
>65	44	40%
<65	66	60%
MTVwb (Median and range, ml)	85.6(3.2–1261.4)	
TLGwb (Median and range, ml)	485.5(12.1–4921.0)	
SUVmaxwb (Median and range)	13.07(3.5–42.9)	

*MTVwb* whole-body metabolic tumor volume, *SUVmaxwb* whole-body maximum standardized uptake value, *TLGwb* whole-body total lesion glycolysis

^a^Other histological subtypes including large cell (*n* = 7), NSCLC not otherwise specified (*n* = 21) and neuroendocrine tumor (*n* = 2)

Univariate Cox regression analysis showed no significant difference in OS on the basis of age, gender, or histology (Table 2). Surgical treatment (24/110 patients) was associated with improved OS, as compared to the no treatment and non-surgical treatment groups. Some of the PET/CT variables, ln(TLG_WB_) and ln(SUV_WB_), were not associated with OS.

**Table 2:** Univariate Cox regression analyses

	Hazard ratio	95% CI	*P*-value
Age (per 1 year increase)	1.02	0 .99 -1.034	0.171
Gender			
Female	Reference		
Male	1.19	0.78–1.83	0.419
Histology			
Adenocarcinoma	Reference		
Squamous	1.37	0.83–2.26	0.218
other types	1.33	0.78 2.27	0.303
Treatment			
Non-surgical (*n* = 76)	Reference		
Surgical (*n* = 24)	0.4	0.22 -0.71	0.002
No treatment (*n* = 10)	0.99	0.47 -2.1	0.986
ln(MTV_wb_)	1.23	1.01–1.49	0.037
ln(TLG_wb_)	1.13	0.95–1.34	0.175
In(SUVmaxwb)	0.91	0.57–1.43	0.67

Note: ln(MTVwb), ln(TLGwb) and ln(SUVmaxwb) are continuous variables. ln = natural logarithmic transformation

However, ln(MTVwb) was positively associated with OS, with a hazard ratio (HR) of 1.23 with 95% confidence interval (95% CI) of 1.01–1.49, (*p* = 0.037). This association persisted on multivariate Cox regression analysis with a HR of 1.28 (95% CI =1.01–1.64, *p* = 0.043), after adjustment for age, gender, treatment and tumor histology (Table 3).

**Table 3:** Multivariate Cox regression analyses of whole cohort

Variables	Hazard ratio	95% CI	*P*-value
ln(MTVwb)	1.28	1.01–1.64	0.043
Treatment			
Non-surgical	Reference		
Surgical	0.41	0.22–0.76	0.004
No treatment	1.34	0.59–3.02	0.48

Additional adjustments were made for age, gender, treatment and tumor histology

**Fig. 2:**
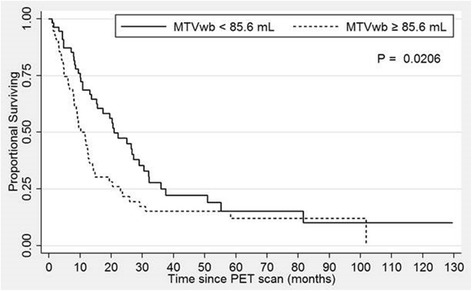
Kaplan-Meier overall survival curves of the training dataset of 110 patients with TNM Stage 3B NSCLC, stratified by the median MTVwb of 85.6 mL. The dashed line indicates patient group with MTVwb greater than or equal to 85.6 ml with median OS of 9.5 months. The solid line indicates patient group with MTVwb less than 85.6 ml with median OS of 17.3 months. The difference is statistically significant with *p* = 0.02

The training dataset was segmented by the median MTVwb of 85.6 mL, Kaplan-Meier survival curves (Fig. 2) demonstrate decreased OS among patients with MTVwb greater than or equal to the median, when compared to those patients with MTVwb less than the median (*p* = 0.021). The median OS was 9.5 months in patients with MTVwb greater than or equal to the median, while the median OS was 17.3 months in patients with MTVwb less than the median.

### Model validation

To validate the model, a separate analysis was performed using a dataset of 44 patients who were imaged and managed from medical center B. These 44 patients received radiation-based treatment. The median OS was 20 months and 1-year, 2-year, and 5-year OS was 60%, 45% and 38%, respectively. 24/44 patients (55%) expired during follow-up. The median follow-up among survivors was 30.7 months (inter-quartile range 12.7 o 49.1 months).

**Fig. 3:**
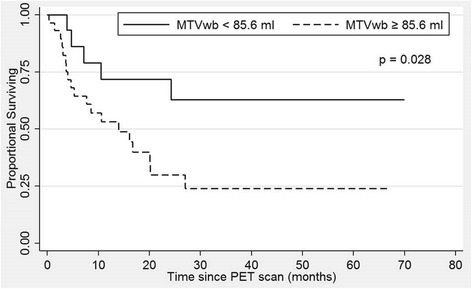
Kaplan-Meier overall survival curves of the validation dataset of 44 patients with TNM stage 3B NSCLC, stratified by the median MTVwb of the training dataset, 85.6 mL. The dashed line represents the patient group with MTVwb ≥85.6 ml with median OS of 13.9 months. The solid line represents the patient group with MTVwb <85.6 ml with median OS not reached during follow-up period. The groups were significantly different with *p* = 0.03

With a univariate Cox regression model, there was significant positive correlation of ln(MTVwb) with OS, HR of 1.83 (95% CI = 1.14–2.94, *p* = 0.012). For 7 of 44 patients, the SUVmax and TLGwb were not available due to missing accurate weight information during PET/CT acquisition. Univariate Cox regression analysis in the remaining 37 patients demonstrated no statistically significant association of ln(TLGwb) (HR = 1.37, 95% CI = 0.94–2.00, *p* = 0.1) and ln(SUVmaxwb) (HR = 1.30, 95% CI = 0.53–3.17, *p* = 0.57) with OS. After adjusting for tumor histology, there remained significant positive correlation with ln(MTVwb) and OS, with HR of 1.78 (95% CI = 1.01–2.90, *p* = 0.02). The results provided further evidence for the prognostic value of ln(MTVwb). Kaplan-Meier survival curves with groups defined by the median MTVwb from the training set (85.6 mL) further demonstrated significantly lower OS among patients with MTVwb ≥85.6 mL, when compared to those with MTVwb <85.6 mL (*p* = 0.028; Fig. 3). The median OS was 13.9 months in patients MTVwb ≥85.6 mL. For patients with MTVwb <85.6 mL, the median OS could not be determined because more than 50% of patients were alive at the last follow-up (median follow-up of 30.7 months).

## Discussion

Quantitative PET/CT parameters are increasingly used to better stratify patients with cancer. Multiple studies have evaluated the association of primary tumor and whole body MTV, TLG, and SUVmax with respect to OS, though this has yet to be evaluated for Stage 3B NSCLC (8^th^ edition TNM classification) (Liao et al., 2012a; Zhang et al., 2013a; Zhang et al., 2013b; Ohri et al., 2015; Liao et al., 2012b; Chung et al., 2014; Im et al., 2015; Hyun et al., 2013; Hyun et al., 2014; Carvalho et al., 2013; Kim et al., 2012; Abelson et al., 2012; Satoh et al., 2014; Winther-Larsen et al., 2016; Yoo et al., 2012; Zhu et al., 2016; Vu et al., 2013; Lee et al., 2007). We sought to better delineate prognosis among patients with Stage 3B NSCLC by utilizing MTVwb, SUVmaxwb, and TLGwb.

Among patients with Stage 3B NSCLC, greater MTVwb was associated with shorter OS, independent of age, sex, and histology. This was further validated by an external dataset of 44 patients treated with radiation-based treatment at outside hospitals. Other studies have shown that OS significantly decreases with every 1 cm increase in primary tumor burden and with increasing number and extent of nodal metastases, which prompted recent changes to the TNM classification (Asamura et al., 2015; Goldstraw et al., 2016). Thus, it is not surprising that quantitative PET/CT measures of whole body tumor burden are similarly associated with OS.

Here we did not find a significant association between SUVmaxwb or TLGwb and OS in patients with Stage 3B NSCLC. SUVmaxwb is a maximum single voxel measurement, so it may not be representative of the entire disease process. Additionally, SUVmaxwb is not representative of the total extent of tumor burden. Though TLGwb incorporates MTVwb, it was not statistically significantly associated with OS in this sample of patients with Stage 3B NSCLC, despite the estimated HR of 1.13. The lack of statistical significance is most likely due to the small sample size in this study, as opposed to a true lack of association with survival.

Among the Stage 3B NSCLC patients in the training cohort, the 2-year and 5-year OS was 34.6% and 13.9%, lower than the survival observed in the IASLC database (44% and 26%, respectively) (Goldstraw et al., 2016). However, our validation dataset had 2- and 5-year survival of 45%, 38%, which is more similar to the IASLC database. One explanation for the difference in OS may be related to socioeconomic status: Center A serves a predominantly low-income neighborhood, a factor that has been previously associated with worse OS (Aldrich et al., 2013). Furthermore, the training dataset included 10 patients who had no treatment, which is likely to lower OS.

The training dataset had a significantly higher proportion of African American patients (55%) than other studies, like the 1996–2007 Florida Cancer Data System registry of 98,541 patients with NSCLC, which was only 7.4% African American (Tannenbaum et al., 2014). Although our study included a disproportionate number of African Americans compared to both other registries and the national population, prior work by Aldrich et al., has shown that lung cancer survival is independent of race, when adjusting for stage and socioeconomic status (Aldrich et al., 2013).

### Limitations

Due to the retrospective design of this study, it was not possible to control the type of surgery, chemotherapy or radiation that patients received. Treatment for each patient was determined at the discretion of the surgeon and oncologist and, ultimately, by the patient. In addition, PET/CT has limited sensitivity to detect lesions which are less than 1 cm in diameter or which have low metabolic uptake, likely resulting in a slight under measurement of MTVwb and TLGwb. Since patients in the validation dataset were exclusively treated with radiation-based treatment, and the significant association between OS and MTVwb persists, this strongly suggests the robust prognostic value of MTVwb.

In summary, among patients with Stage 3B NSCLC increasing MTVwb measured on baseline FDG PET/CT is associated with worse OS in both univariate and multivariate cox regression analysis, as seen in both the training and validation datasets. Currently, PET/CT volumetric parameters do not affect clinical management, but hopefully the developing knowledge regarding the values of these measurements may allow more targeted treatment for patients with more or less aggressive disease. Regardless, identifying which patients are more likely to survive to 2 or 5 years may be extremely valuable to patients, who may be planning the final months or years of their life.

## Conclusion

MTVwb is a prognostic marker for OS in patients with Stage 3B NSCLC, independent of age, gender, treatment, and tumor histology.

## Abbreviations

AJCC: American Joint Committee on Cancer
CT: Computed tomography
FDG: 2-deoxy-2-[18F]fluoro-D-glucose
HR: Hazard ratio
IASLC: International Association for the Study of Lung Cancer
MBq: Megabecquerel
MTVwb: Whole-body metabolic tumor volume
NSCLC: Non-small cell lung cancer
OS: Overall survival
PET: Positron emission tomography
SUVmaxwb: Maximum standardized uptake value
TLGwb: Whole body total lesion glycolysis
TNM: Tumor-node-metastases
UICC: Union for International Cancer Control
